# Chemical Characteristics and Antioxidant Activity of *Arctostaphylos uva-ursi* L. Spreng. at the Southern Border of the Geographical Range of the Species in Europe

**DOI:** 10.3390/molecules26247692

**Published:** 2021-12-20

**Authors:** Piotr Sugier, Łukasz Sęczyk, Danuta Sugier, Rafał Krawczyk, Małgorzata Wójcik, Joanna Czarnecka, Sylwia Okoń, Andrzej Plak

**Affiliations:** 1 Department of Botany, Mycology and Ecology, Institute of Biological Sciences, Maria Curie-Skłodowska University, 19 Akademicka Street, 20-033 Lublin, Poland; piotr.sugier@mail.umcs.pl (P.S.); joanna.czarnecka@mail.umcs.pl (J.C.); 2 Department of Industrial and Medicinal Plants, University of Life Sciences in Lublin, 15 Akademicka Street, 20-950 Lublin, Poland; lukasz.seczyk@up.lublin.pl; 3 Department of Zoology and Nature Protection, Institute of Biological Sciences, Maria Curie-Skłodowska University, 19 Akademicka Street, 20-033 Lublin, Poland; rafal.krawczyk@mail.umcs.pl; 4 Department of Plant Physiology and Biophysics, Institute of Biological Sciences, Maria Curie-Skłodowska University, 19 Akademicka Street, 20-033 Lublin, Poland; malgorzata.wojcik@mail.umcs.pl; 5 Institute of Plant Genetics, Breeding and Biotechnology, University of Life Sciences in Lublin, 15 Akademicka Street, 20-950 Lublin, Poland; sylwia.okon@up.lublin.pl; 6 Institute of Earth and Environmental Sciences, Maria Curie-Skłodowska University, Kraśnicka Av. 2d, 20-718 Lublin, Poland; andrzej.plak@mail.umcs.pl

**Keywords:** bearberry, arbutin, natural antioxidants, heathlands, pine forests

## Abstract

The bearberry (*Arctostaphylos uva-ursi* L. Spreng.) is a source of herbal material—bearberry leaf (*Uvae ursi folium*), which is highly valued and sought by pharmaceutical and cosmetic industries. For many years, leaves of this plant have been used in traditional medicine as a diuretic, antimicrobial, and anti-inflammatory agent for various diseases of the urogenital tract. The bearberry has also been proposed as a natural antioxidant additive due to the high contents of phenolic compounds in its leaves. The study was focused on characterization of the basic phytochemical composition and antioxidant activity of extracts derived from bearberry leaves collected from plants located at the southern border of the geographical range of the species in Europe. The investigated herbal material is characterized by a different chemical profile compared to the chemical profiles of bearberry found in other parts of the continent. Bearberry extracts from plants growing in two different habitat types—heathlands and pine forests showed a wide range of variation, especially in the concentration of hyperoside, corilagin, and methylartutin and the total flavonoid contents. In addition to arbutin, bearberry can be a valuable source of phenolic compounds, which are mainly responsible for the antioxidant properties of extracts. The high content of phenols and high values of antioxidant parameters indicate a high potential of bearberry leaves to be used as a powerful natural source of antioxidants in herbal preparations. Therefore, the *A. uva-ursi* populations can be a source of plant material for pharmaceutical, cosmetic, and food industries.

## 1. Introduction

Many Ericaceae plant species have a long history of ethnopharmacological use and are important for medicinal and pharmacological purposes [1]. This family is represented, e.g., by the bearberry (*Arctostaphylos uva-ursi* L. Spreng.), which has been used in folk medicine for centuries as a rich source of raw material abundant in secondary metabolites. This plant species is an evergreen dwarf shrub. It can be found at high and mid-latitudes in Europe, Asia, and North America. This circumpolar plant reaches the southern limit of its compact range in the south of Poland [2]. In southern and south-western Europe, the bearberry is noted in mountainous regions [3,4,5]. In Central Europe, it occurs mainly in the lowlands—in pine forests on podsolic soils and heathlands situated on sandy dunes [6].

In the last decades, the use of medicinal plants has been growing rapidly in the world due to the increasing demand for natural health products, herbal drugs, and secondary metabolites [7,8]. Natural products and their derivatives are important sources of novel therapeutic substances [9]. Therefore, widespread pressure to obtain plant material from areas of natural occurrence is observed; however, it is very often limited by the legal restrictions of conservation and sustainable use of medicinal plants [8,10,11]. At the same time, new sources of secondary metabolites are widely being searched [12,13], and plant cultivation trials are carried out to obtain enough raw material with a new chemical profile in controlled conditions [13,14]. The present study focuses on the bearberry, i.e., an endangered species in Europe [15] included in the European red list of medicinal plants [16]. Presently, this species is under strict law protection and is placed on the red lists in Poland, the Czech Republic, Slovakia, and Bulgaria [17,18,19]. Moreover, investigations have shown that harvesting aerial biomass in unprotected areas decreases the regenerative capacity of *A. uva-ursi* populations [20,21]. Therefore, recommendations or guidelines for harvesting as part of the sustainable management of the population of this species in natural habitats have been formulated [22]. It should be mentioned that, in Europe, the need to acquire new chemotypes of herbal plants is emphasized as the most effective way to meet the industry’s demand for raw material and to reduce the pressure exerted by harvesting in countries where it is possible to obtain material from natural populations.

The bearberry is a source of herbal material—bearberry leaf (*Uvae ursi folium*), which is highly valued and sought by pharmaceutical and cosmetic industries both in Poland and in Europe [23,24]. During the last years, the chemical composition of bearberry leaves has been intensively studied [25,26,27]. It has been found that *Uvae ursi folium* is a rich source of phytochemicals, especially phenolic compounds. Phenolic glycoside arbutin is considered the main bioactive compound of this plant. Moreover, the chemical profile of bearberry leaves is characterized by the presence of ursolic acid, tannic acid, gallic acid, p-coumaric acid, syringic acid, galloylarbutin, gallotannins, and glycosidic forms of flavonoids, e.g., quercetin, kaempferol, and myricetin glycosides, penta-O-galloyl-β-d-glucose, hyperoside, corilagin, and picein [1,25,26,27,28]. The health benefits of the bearberry are provided by the compounds comprised in its valuable composition.

For many years, leaves of this plant have been used in traditional medicine as a diuretic, antimicrobial, and anti-inflammatory agent for various diseases of the urogenital tract [27]. *Arctostaphylos uva-ursi* leaf extracts are characterized by antioxidant [4,29] and antimicrobial activity [30,31] and have been used as skin-whitening agents and antioxidant agents in food and food packaging formulations [4,32,33]. In recent decades, many studies have shown that bearberry leaf extracts have strong antibacterial activity against bacterial strains that cause urinary tract infections [34]. They are remedies for several diseases, including diuresis [35,36] and have antiproliferative effects against human carcinoma cell lines [37].

The accumulation of secondary metabolites is strongly dependent on genetic, ontogenic, morphogenetic, and various environmental factors such as light, temperature, soil water capacity, soil fertility, and salinity. Changes in one factor may alter the content of secondary metabolites even if the other factors remain constant [38]. In literature, there are reports of diversified and changeable responses of various plant species to different environmental stresses in terms of production of secondary metabolites [38]. In the case of the bearberry from the Iberian Peninsula, such factors as altitude, UV-radiation, the date of collection, and location of plants have been proven to influence the chemical composition [3,4,5]. Therefore, it can be assumed that the acid and poor sandy soils, characteristic vegetation, and climatic conditions at the southern border of the geographical range of the species in Europe can affect the chemical profile in the bearberry and, in consequence, the antioxidant activity of its extracts.

**Figure 1:**
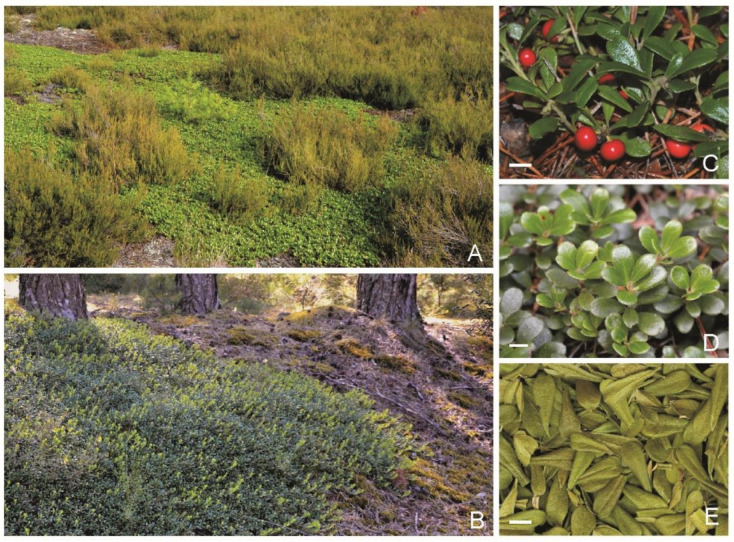
*Arctostaphylos uva-ursi*. (**A**)—heathland population, (**B**)—pine forest population, (**C**)—fruiting individual, (**D**)—one-year-old shoots, (**E**)—raw material; lines indicate a length of 1 cm.

Generally, there are scarce investigations of the chemical profiles in bearberry populations occurring in natural habitats in Europe. In many studies on the chemistry of the raw material and biological activity, raw material from pharmacies, supermarkets, and collections was used. In Europe, very detailed characteristics, such as the chemical composition and biological activity, have recently been presented from the Iberian Peninsula in the context of selection of plant material for pharmaceutical, cosmetic, and food industries [4,5], where plant material was collected from the typical bearberry habitats located in mountain communities (424–1410 m a. s. l.). Conversely, the *A. uva-ursi* populations selected in this study come from extremely and harsh sites situated at the southern border of the dense geographical range of the species in Europe. This is the first analysis of raw material originating from populations growing in completely different conditions (acid and poor sandy soils of heathlands and pine forests) (Figure 1). It is very often observed that stress and disturbances in harsh habitat conditions affect the response of plants through accumulation of secondary metabolites [38]. This yields plant material with a valuable chemical composition, which can exhibit new properties and, in consequence, new activities that can be used in medicine, pharmacy, or cosmetic industry. In the case of the Polish populations of *A. uva-ursi*, the knowledge of the chemical characteristics and biological activity of leaves is still insufficient. Therefore, the objective of this study was to characterize the basic phytochemical composition and antioxidant activity of extracts derived from bearberry leaves collected from plants located at the southern border of the geographical range of the species in Europe. The results presented in this paper can contribute to appropriate selection of plant material for pharmaceutical, cosmetic, and food industries.

## 2. Results and Discussion

### 2.1. Characteristics of Secondary Metabolites

**Figure 2:**
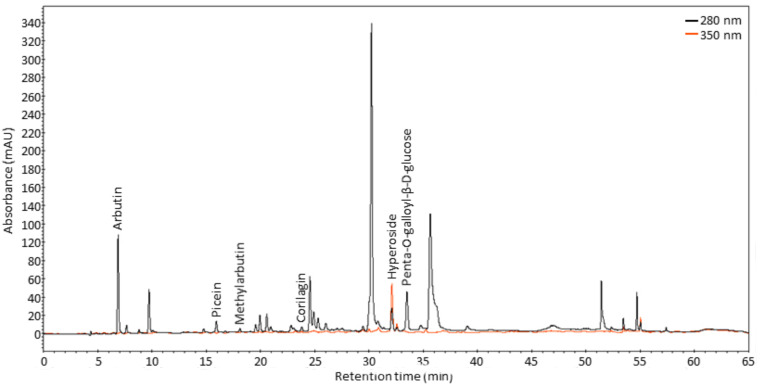
HPLC chromatograms of bearberry ethanolic extract at 280 nm (black line) and 350 nm (orange line).

Arbutin, methylarbutin, penta-O-galloyl-β-d-glucose, hyperoside, picein and corilagin contents were determined using high-performance liquid chromatography (HPLC). Examples of chromatograms of *A. uva-ursi* phytochemicals are presented in Figure 2.

**Figure 3:**
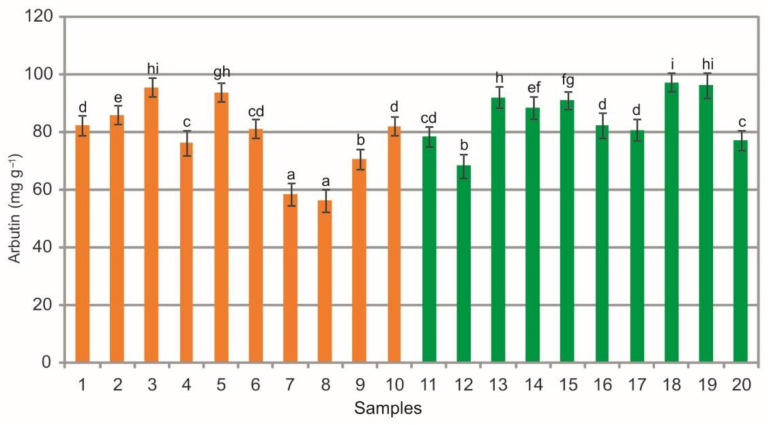
Arbutin contents (mg g^−1^) in leaf extracts from bearberry plants collected in heathland populations (orange) and in pine forest populations (green). For each site, data are mean and SD. The values designated by the different letters are significantly different (Tukey test, *p* < 0.05).

The analysis of extracts from the bearberry leaves showed a wide phytochemical variation in the arbutin (ARB) content, i.e., from 56.32 mg g^−1^ to 95.55 mg g^−1^ in samples collected in the heathlands and from 68.33 mg g^−1^ to 97.26 mg g^−1^ in the case of samples collected in the pine forests (Figure 3). There are differences in the mean values of this substance between the samples collected in the heathland, the samples from the pine forest, and between samples from both plant communities (ANOVA, F = 510.3, *p* < 0.01, Figure 3).

Arbutin is a natural component of *Ericaceae* such species as *Vaccinium myrtillus*, *V. vitis-idaea*, and *V. uliginosum*. The bearberry has the highest content of this compound [1]. According to the European Pharmacopoeia [24], the ARB content in dried leaves of *A. uva-ursi* must be at least 70 mg g^−1^ to recognize the plant material as herbal. The above-mentioned research results indicate that, in most cases, the bearberry leaves meet the Pharmacopeia requirements regarding the arbutin content; however, some samples of leaves, especially those originating from the heathland populations, cannot be classified as herbal material. Similarly, low content of ARB (59.5 mg g^−1^) was reported by Matsuda et al. [39] and Parejo et al. [3] in four natural populations growing at different altitudes in the Catalan Pyrenees (Spain), where the level of arbutin in the studied populations ranged from 6.3% to 9.1%. The content of this metabolite in the raw material from the heathland and pine forest sites is lower than the concentration in mountain populations in the Iberian Peninsula, where it ranged between 87.1 mg g^−1^ and 211.5 mg g^−1^ [5]. Asensio et al. [5] demonstrated higher arbutin contents in leaves of bearberry plants growing in northern locations and at relatively higher altitudes characterized by lower mean temperatures, higher annual precipitations, and lower global radiation levels. In turn, Panusa et al. [26] analyzed commercial samples labeled “bearberry leaf” with different origin and reported a concentration of arbutin of 85.0 mg g^−1^. As shown by Olennikov and Chekhirova [25], samples of *A. uva-ursi* from Buryatiya (Asiatic part of Russia) contained 82.40 mg g^−1^ of arbutin.

**Figure 4:**
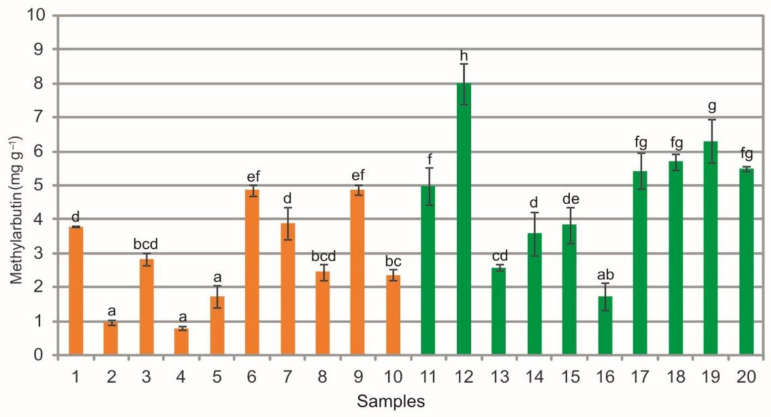
Methylarbutin contents (mg g^−1^) in leaf extracts from bearberry plants collected in heathland populations (orange) and in pine forest populations (green). For each site, data are mean and SD. The values designated by the different letters are significantly different (Tukey test, *p* < 0.05).

The analysis of extracts from the bearberry leaves revealed a wide phytochemical variation in the content of methylarbutin (mARB), i.e., from 0.80 mg g^−1^ to 4.88 mg g^−1^, in samples collected in the heathlands and from 1.74 mg g^−1^ to 8.00 mg g^−1^ in samples collected in the pine forests (Figure 4). Differences in the mean values of this substance can be seen between the samples from the heathland, the samples collected in the pine forest, and between samples representing both plant communities (ANOVA, F = 80.5, *p* < 0.01, Figure 4).

In many cases the extracts from bearberry leaves collected from the pine forest populations are characterized by significantly higher content of mARB in comparison with the heathland populations (Figure 4). There are scarce literature data on the content of this compound. However, in comparison with the values reported by Barl et al. [28], i.e., up to 4% of this compound in extracts of bearberry leaves originating from Canada, the content of this metabolite in the raw material analyzed in the present study is very low (Figure 4).

**Figure 5:**
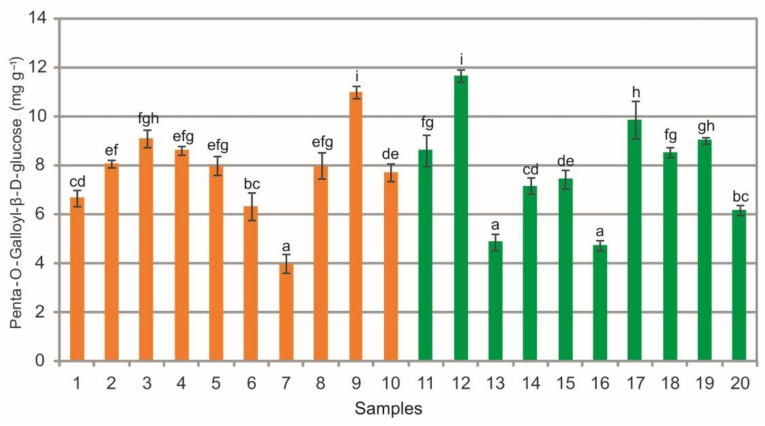
Penta-O-galloyl-β-d-glucose contents (mg g^−1^) in leaf extracts from bearberry plants collected in heathland populations (orange) and in pine forest populations (green). For each site, data are mean and SD. The values designated by the different letters are significantly different (Tukey test, *p* < 0.05).

The extracts from the bearberry leaves collected in the heathlands exhibited a wide phytochemical variation in the penta-O-galloyl-β-d-glucose (PGG) content, which ranged from 3.99 mg g^−1^ to 11.02 mg g^−1^, similar to the extracts from the leaves collected in the pine forests, i.e., from 4.75 mg g^−1^ to 11.68 mg g^−1^ (Figure 5). Differences in the mean values of this substance were noted within the heathland sample group, pine forest sample group, and between samples representing both plant communities (ANOVA, F = 97.6, *p* < 0.01, Figure 5). These are low contents (ca. 50%) in comparison with the results obtained by Olennikov and Chekhirova [25], who determined PGG also in *A. uva-ursi* shoots in the range of 12.14–21.89 mg g^−1^ depending on the raw material sample. Penta-O-galloyl-β-d-glucose, i.e., gallotannin, is a polyphenolic compound occurring naturally in several medicinal plants: *Paeonia suffruticosa* [40], *Rhus chinensis* [41], *Acer truncatum* [42], *Schinus terebinthifolius* [43], *Terminalia chebula* [44], *Fomitella fraxinea* [45], *Rhus verniciflua* [46], and *Oenothera paradoxa* [47]. Several in vitro and some in vivo studies have shown that PGG exhibits multiple biological activities indicating a great potential for PGG to be used in the therapy and prevention of several major diseases, including cancer and diabetes [42,48,49,50,51]. Moreover, recent studies have shown that PGG inhibits TNF-α-activated CXCL1/GRO-α expression and induces apoptosis-related genes in triple-negative breast cancer cells [52]. In turn, the results of studies on *Fomitella fraxinea* indicate that PGG exerts anti-photoaging effects in vitro and in vivo by the suppression of PAK1 and JNK1 kinase activities and may be useful for the prevention of skin aging [46].

**Figure 6:**
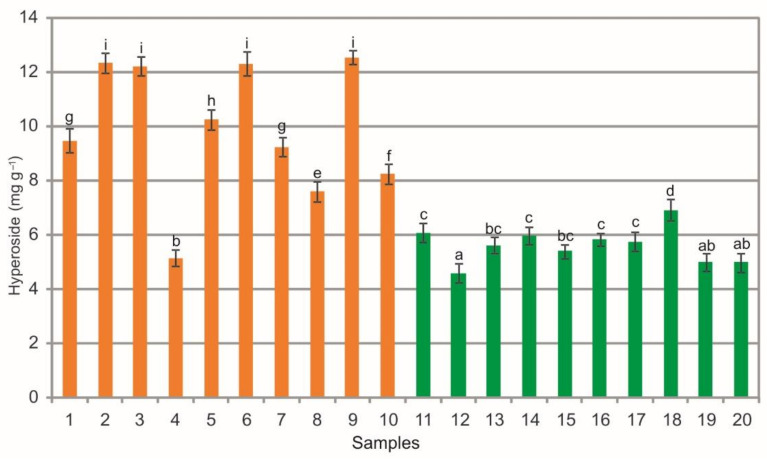
Hyperoside contents (mg g^−1^) in leaf extracts from bearberry plants collected in heathland populations (orange) and in pine forest populations (green). For each site, data are mean and SD. The values designated by the different letters are significantly different (Tukey test, *p* < 0.05).

The analysis of extracts from the bearberry leaves collected in the heathlands showed a wide phytochemical variation in the hyperoside (HYP) content ranging from 5.16 mg g^−1^ to 12.55 mg g^−1^. Similarly, the extracts from the leaves collected in the pine forests contained from 4.61 mg g^−1^ to 6.93 mg g^−1^ of HYP (Figure 6). There are differences in the mean values of this substance between the heathland samples, the samples collected in the pine forest, and between samples representing both plant communities (ANOVA, F = 1032.3, *p* < 0.01, Figure 6). In most cases the extracts from bearberry leaves collected from the heathland populations are characterized by significantly higher content of HYP in comparison with the pine forest populations (Figure 6). Hyperoside is a flavonoid occurring naturally in several medicinal plants. It is most often extracted from *Hypericum* spp. and *Crataegus* spp. This metabolite shows broad-spectrum biological and therapeutic activities, e.g., antioxidant [53,54] anti-inflammatory [55], and anticancer effects [53]. The HYP concentrations in the extracts analyzed in this study are generally high; they are several times higher (in the case of the leaves collected in the heathlands) than the values (2.92 mg g^−1^) reported by Olennikov and Chekhirova [25]. The HYP concentrations obtained from the leaves collected in the heathlands are similar to values shown in raw material of *Hypericum perforatum*, which is a hyperoside-rich species (15.5 mg g^−1^), by Zdunic et al. [56]. Some bearberry samples were characterized by the content of hyperoside >7 mg g^−1^. This hyperoside content is required for *Hyperici herba* according to the Polish Pharmacopoeia [23].

**Figure 7:**
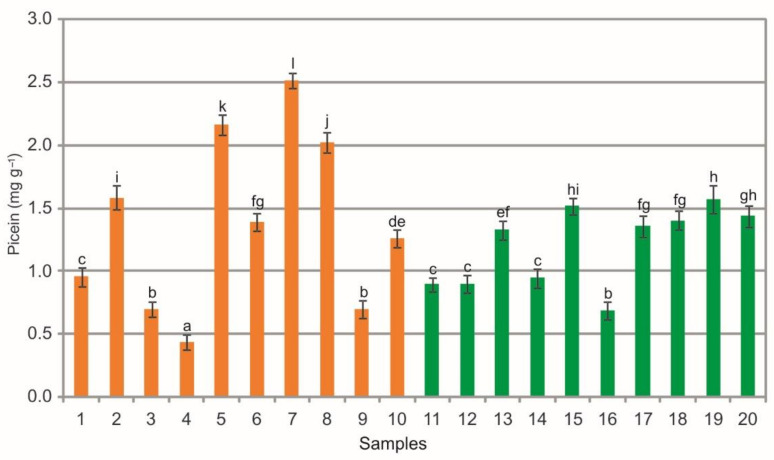
Picein contents (mg g^−1^) in leaf extracts from bearberry plants collected in heathland populations (orange) and in pine forest populations (green). For each site, data are mean and SD. The values designated by the different letters are significantly different (Tukey test, *p* < 0.05).

The extracts from the bearberry leaves collected in the heathlands were characterized by a wide phytochemical variation in the picein (PIC) content, which ranged from 2.51 mg g^−1^ to 50.44 mg g^−1^, as in the case of extracts from the leaves collected in the pine forests (from 0.69 mg g^−1^ to 1.57 mg g^−1^) (Figure 7). Differences in the mean values of this substance are visible between the samples collected in the heathland, the pine forest samples, and between samples from both plant communities (ANOVA, F = 1039.7, *p* < 0.01, Figure 7). Picein is a phenolic compound commonly found in *Picea abies* and some shrub species. Picein isolated from hot water extract of willow bark has been found to act as a natural antioxidant secondary metabolite and can play a role as a potent neuroprotectant [57]. The picein content in the bearberry leaves obtained in the present study is very low in relation to its several times higher content in leaves (12.32 mg g^−1^) reported by Olennikov and Chekhirova [25]. Among phenolic compounds, picein was the dominant component of bearberry stems with a concentration up to 68.12 mg g^−1^ [25].

**Figure 8:**
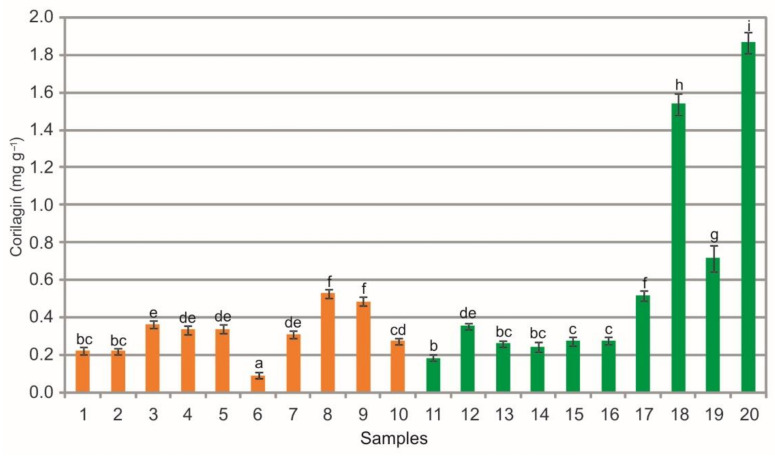
Corilagin contents (mg g^−1^) in leaf extracts from bearberry plants collected in heathland populations (orange) and in pine forest populations (green). For each site, data are mean and SD. The values designated by the different letters are significantly different (U Mann-Whitney test, *p* < 0.05).

The analysis of extracts from the bearberry leaves collected in the heathlands showed a wide phytochemical variation in the corilagin (COR) content ranging from 0.09 mg g^−1^ to 0.53 mg g^−1^, likewise the extracts from the leaves collected in the pine forests (from 0.19 mg g^−1^ to 1.87 mg g^−1^) (Figure 8). The statistical analysis showed statistically significant differences between the populations. Differences in the mean values of this substance were noted within the heathland sample group, pine forest sample group, and between samples representing both plant communities (Kruskal-Wallis test, H = 58.4, *p* < 0.001, Figure 8). In many cases the extracts from bearberry leaves collected from the pine forest populations are characterized by significantly higher content of COR in comparison with the heathland populations (Figure 8). Corilagin is a natural plant polyphenol representing ellagitannins. It is the major active component of many ethnopharmacological plants such as *Phyllanthus emblica, P. niruri*, and *P. urinaria* [58,59,60]. COR shows broad-spectrum biological and therapeutic activities, e.g., antioxidant [61], anti-inflammatory [62], and hepatoprotective effects [63]. This molecule has inhibitory activity against the growth of numerous cancer cells [64] and is a promising medicinal herbal agent [65]. The COR concentration in the bearberry leaves from the Polish populations is very low in comparison with the Asiatic population [25], which exhibited a several times higher level of COR in leaves (up to 57.51 mg g^−1^).

No myricetin was detected in the chemical analyses of the bearberry extracts. This metabolite shows radical scavenging activity [66]. The absence of this metabolite is in agreement with the reports by Olennikov and Chekhirova [25], who did not observe myricetin and its derivative in samples of *A. uva-ursi* from Buryatiya (Asiatic part of Russia). A similar situation was observed by Panusa et al. [26], who analyzed commercial samples labeled “bearberry leaf” with different origins. The authors proposed that myricetin, together with a galloyl arbutin isomer and a disaccharide, are distinctive metabolites in another plant species—*Arctostaphylos pungens*. On the contrary, in Spanish populations, the content of this metabolite was even 17.2 mg g^−1^, and a higher level of this compound was detected in bearberry plants growing in locations with higher radiation levels [5]. Myricetin was also observed in bearberry leaves collected from the boreal forest of the James Bay region of northern Quebec in Canada [67].

**Figure 9:**
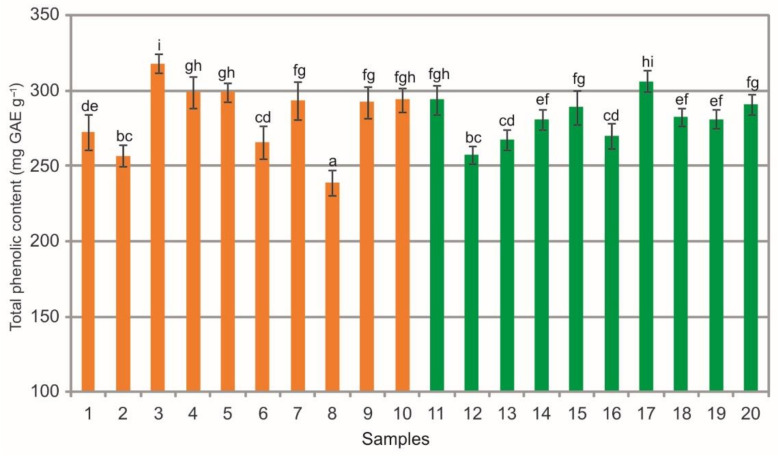
Total phenolic content (mg GAE g^−1^) contents in leaf extracts from bearberry plants collected in heathland populations (orange) and in pine forest populations (green). For each site, data are mean and SD. The values designated by the different letters are significantly different (U Mann-Whitney test, *p* < 0.05).

The extracts from the bearberry leaves collected in the heathlands exhibited a significant phytochemical variation in total phenolic content (TPC), which ranged from 238.85 mg GAE g^−1^ to 318.28 mg GAE g^−1^; similarly, in the case of extracts from the leaves collected in the pine forests, TPC ranged from 257.51 mg GAE g^−1^ to 306.57 mg GAE g^−1^ (Figure 9). The statistical analysis showed statistically significant differences between the populations. Differences in the mean values of this TPC were noted within the heathland sample group, pine forest sample group, and between samples collected in both plant communities (Kruskal-Wallis test, H = 55.9, *p* < 0.001, Figure 9). The TPC in the bearberry leaves is higher to that determined in Spain populations, where the mean values of this parameter oscillated in the range of 170–180 mg GAE g^−1^. In many cases, the TPC in the extracts analyzed in this study was twofold higher than in bearberry extracts from the Iberian Peninsula [5].

**Figure 10:**
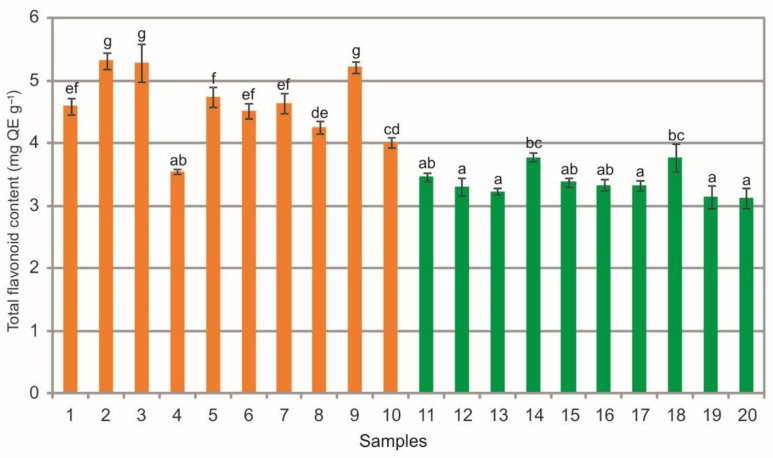
Total flavonoid content (mg QE g^−1^) contents in leaf extracts from bearberry plants collected in heathland populations (orange) and in pine forest populations (green). For each site, data are mean and SD. The values designated by the different letters are significantly different (Tukey test, *p* < 0.05).

The analysis of extracts from the bearberry leaves collected in the heathlands showed a wide phytochemical variation in total flavonoid content (TFC) ranging from 3.55 mg QE g^−1^ to 5.32 mg QE g^−1^, as in the case of extracts from the leaves collected in the pine forests, i.e., from 3.13 mg QE g^−1^ to 3.78 mg QE g^−1^ (Figure 10). The statistical analysis showed statistically significant differences between the populations. There are differences in the mean values of this substance between the samples collected in the heathland, between the samples from the pine forest, and between samples representing both plant communities (ANOVA, F = 1039.7, *p* < 0.01, Figure 10). In most cases, the extracts from bearberry leaves collected from the heathland populations are characterized by significantly higher content of TFC in comparison with the pine forest populations (Figure 10). It should be mentioned that measured content and activity of potentially active phytochemicals in bearberry, and consequently in its extracts, depended on many factors, e.g., genetic variation, growing conditions (occurrence), post-harvest treatment, and extraction and analysis methods [5,25,27,29,33,36].

Figure 9 shows the results of the PCA ordination of the bearberry samples. The eigenvalues of the first (2.56), second (1.55), and third axes (1.42) indicate the presence of three gradients, within which the samples are differentiated in terms of the chemical composition (Table 1). The first three axes explain 69.13% of the variability (31.96% the first axis, 19.38% the second axis, 17.78 the third axis), which proves that the traits correlated with these axes are greatly important for interpreting the differentiation and correlations between these secondary metabolites. The content of quercetin and hyperoside in the bearberry extracts are clearly positively correlated with the first axis, whereas mARB, PGG, and COR are negatively correlated. The content of PGG and TPC are positively correlated with the second axis. In turn, the content of PIC, COR, and TPC are clearly positively correlated with the third axis, whereas ARB and PGG are negatively correlated. The first axis presents a gradient along which an increase in the concentration of quercetin and hyperoside and a decrease in the concentration of mARB and COR are observed, which implies that primarily the concentration of these metabolites determines the differentiation of the chemical composition of the examined extracts. The variation between the study samples is evident as well. Two groups can be distinguished in the ordination space of PCA: a group of samples taken from the heathland populations and a group of samples taken from the pine forest populations. The former heathland populations are characterized by the highest content of TFC and HYP and the lowest content of mARB and COR in relation to the latter group.

**Figure 11:**
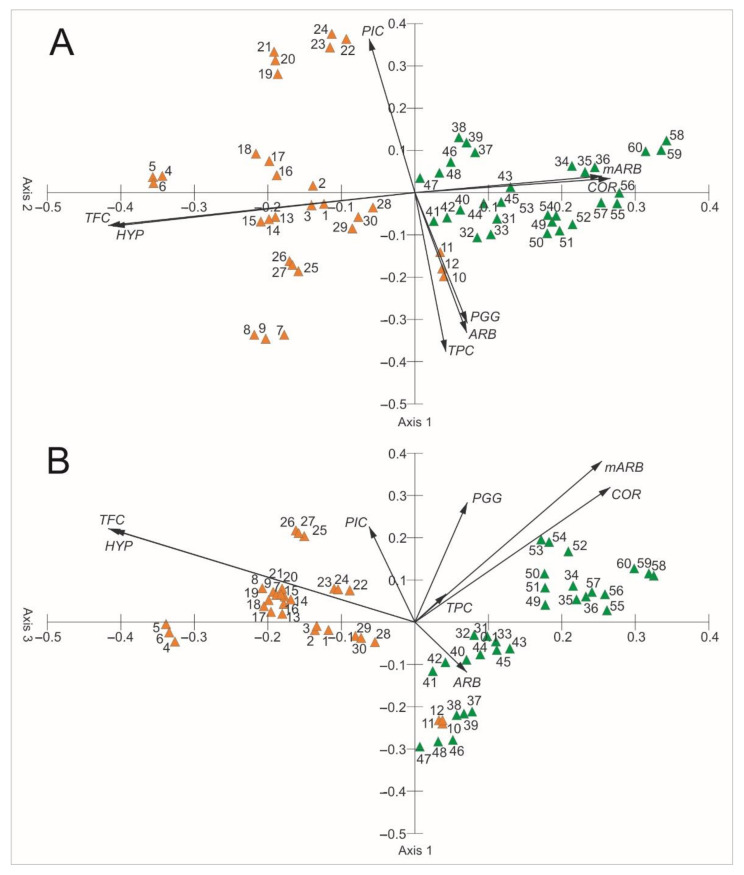
Results of PCA based on the chemical composition of bearberry leaf extracts. A—Axis 1 and Axis 2, B—Axis 1 and Axis 3, ARB—arbutin, mARB—methylarbutin, PGG—penta-O-galloyl-β-d-glucose, HYP—hyperoside, PIC—picein, COR—corilagin, TPC—total phenolic content, TFC—total flavonoid content.

The comparison of the values of particular parameters between the two groups (heathlands vs. pine forests) underlines the difference in the chemical composition of the analyzed extracts (Figure 3, Figure 4, Figure 5, Figure 6, Figure 7, Figure 8, Figure 9, Figure 10 and Figure 11, Table 1). The extracts from bearberry leaves collected from the heathland populations are characterized by significantly higher content of TFC and HYP and significantly lower content of mARB and COR in comparison with the pine forest population. The present results show that the type of vegetation and the related variable species composition and light conditions can modify the chemical composition or bearberry raw material, likewise the altitude, precipitations, and radiation [5].

### 2.2. Antioxidant Activity

In recent years, a growing interest in the production of natural has been observed [68,69,70]. Therefore, as an alternative to synthetic chemicals, plant-derived substances, with special emphasis on natural antioxidants, are being sought. Antioxidant phytochemicals are generally regarded as safe in use and commonly acceptable, thus their application in, e.g., pharmaceuticals, cosmetics, foods, and natural food packages has received increased research attention [68,69,70,71]. To determine the antioxidant potential of bearberry, basic antioxidant tests—ABTS^•+^ and scavenging activity, ferric reducing power, and ferrous chelating ability were performed.

The results of the analysis showed a variation in the antioxidant activity parameters in the studied extracts (Table 2). The range of the ABTS parameter was from 530.63 mg TE g^−1^ to 713.49 mg TE g^−1^ and from 543.71 mg TE g^−1^ to 652.70 mg TE g^−1^ for samples collected in the heathlands and the pine forests, respectively. The variations in DPPH ranged from 342.38 mg TE g^−1^ to 756.62 mg TE g^−1^ and from 377.35 mg TE g^−1^ to 518.34 mg TE g^−1^ for samples collected in the heathlands and the pine forests, respectively. The range of the RP parameter was from 343.38 mg TE g^−1^ to 408.05 mg TE g^−1^ and from 351.89 mg TE g^−1^ to 402.67 mg TE g^−1^ for samples collected in the heathlands and the pine forests, respectively. The variations in CHEL ranged from 0.06 mg EDTA g^−1^ to 0.32 mg EDTA g^−1^ and from 0.04 mg EDTA g^−1^ to 0.28 mg EDTA g^−1^ for samples collected in the heathlands and the pine forests, respectively. There were differences in the mean values of this substance within the heathland sample group, pine forest sample group, and between samples from both plant habitats (ABTS: H = 55.7, *p* < 0.001; DPPH: F = 117.2, *p* < 0.01; RP: F = 35.8, *p* < 0.01; CHEL: H = 55.02, *p* < 0.01; Table 2). Trolox is a water-soluble synthetic analogue of vitamin E (alpha-tocopherol) with very strong and well documented antioxidant properties; therefore, it is commonly used in biochemical studies and as a standard in antioxidant assays [72,73]. The very high value obtained after expression of the results in Trolox equivalent and the corresponding high activity of compounds occurring naturally in the bearberry leaves indicates that these phytochemicals are very strong antioxidants, even in comparison with synthetic antioxidants. Furthermore, compared with other valuable natural sources of antioxidants, e.g., Greek oregano, whose extracts and by-products are recognized as strong antioxidant agents for food preservation [74], higher antioxidant activity was determined for bearberry. Previous studies indicate that the antiradical activity in Greek oregano ranged from 150.8 to 235.1 mg TE g^−1^ against ABTS, from 101.8 to 160.7 mg TE g^−1^ against DPPH, and from 73.2 to 114.4 TE g^−1^ of ferric reducing power, depending on cultivation conditions (nitrogen dose), in the case of the same analytical procedures [75].

**Figure 12:**
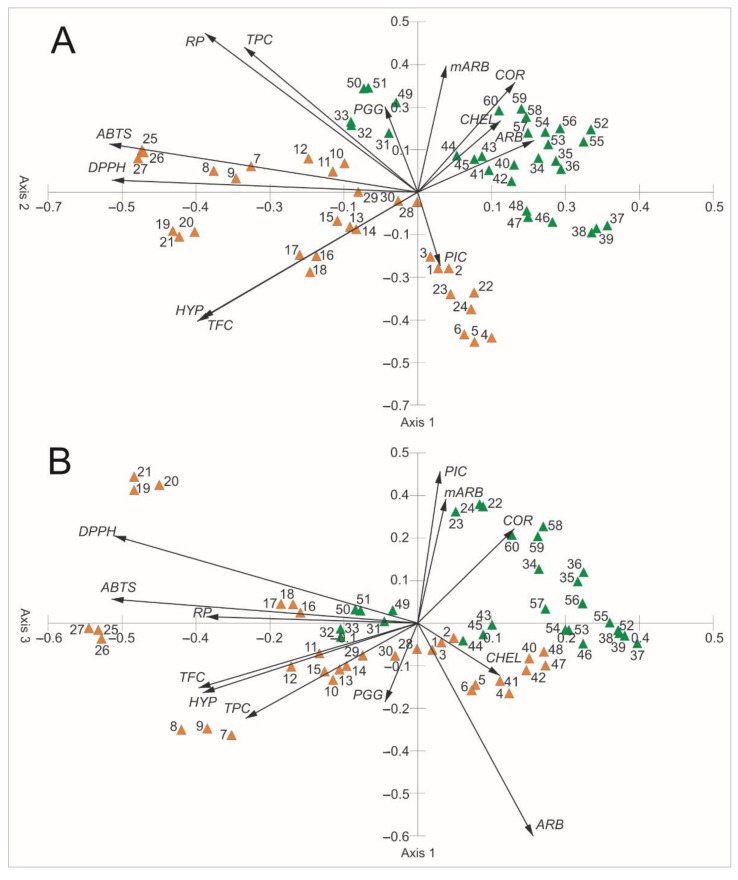
Results of PCA based on the chemical composition of bearberry leaf extracts and antioxidant activity parameters. A—Axis 1 and Axis 2, B—Axis 1 and Axis 3, ARB—arbutin, mARB—methylarbutin, PGG—penta-O-galloyl-β-d-glucose, HYP—hyperoside, PIC—picein, COR—corilagin, TPC—total phenolic content, TFC—total flavonoid content, ABTS—ABTS^•+^ scavenging activity, DPPH—DPPH^•^ scavenging activity, RP—reducing power, CHEL—chelating ability.

Figure 12 shows the results of the PCA ordination of the bearberry samples. The eigenvalues of the first (3.38), second (2.48), and third axes (1.60) indicate the presence of three gradients, within which the samples are differentiated in terms of the chemical composition (Table 3). The first three axes explained 62.15% of the variability (28.17% the first axis, 20.65% the second axis, 13.32 the third axis), which proves that the traits correlated with these axes are greatly important for interpreting the differentiation and correlations between these secondary metabolites. All antioxidant activity parameters are negatively correlated with axis 1 (Figure 12, Table 3). ABTS, RP, and CHEL are positively correlated with axis 2, whereas TFC is negatively correlated. DPPH is positively correlated with axis 3, whereas CHEL is negatively correlated. There are two groups of samples in the ordination space representing the heathland populations and the pine forest populations (Figure 12, Table 3). The samples from the heathland populations create a looser group and are located in the lower left part of the ordination space. The correlation between secondary metabolites and antioxidant activity parameters shows an impact of the content of ARB on DPPH, mARB on ABTS, PGG, HYP, and COR on EDTA, TPC on ABTS and RP, and TFC on CHEL (Figure 12, Table 4). In turn, the samples from the pine forest populations created a more compact group and are located in the upper right part of the ordination space. The correlation between secondary metabolites and antioxidant activity parameters inside this group show the impact of the content of ARB on DPPH, PGG on ABTS, HYP on CHEL, TPC on ABTS, DPPH, and RP, and TFC on CHEL. A negative correlation between mARB, PIC, COR, and CHEL was observed (Figure 12, Table 5).

Presently, there are scarce literature data on the influence of environmental factors on the chemical profile in the bearberry [5]. The present study demonstrated the chemical profiles in the bearberry raw material differing in the ARB, MIR, and TPC content from the profile in the Iberian Peninsula population [5] and in the PIC content from the Russian population [25]. Additionally, the influence of habitat conditions on the chemical composition in bearberry leaves and the activity of the tested plant extracts.

## 3. Materials and Methods

### 3.1. Habitat Characteristics and Plant Material

The field study was carried out in August 2020. Twenty populations consisting of several tens of individuals, and 10 populations present in heathlands (1–4: 50°26′27″ N; 21°48′59″ E; 5–8: 50°42′06″ N; 22°01′19″ E; 9: 50°43′47″ N; 22°00′54″ E; 10: 50°45′05″ N; 22°06′25″ E), and 10 populations from a pine forests (11–17: 51°25′18″ N; 23°35′16″ E; 18–20: 51°45′27″ N; 22°11′45″ E) were analyzed. The populations were representative of the natural distribution of this plant species at the southern border of its geographical range in Europe. The bearberry forms dense carpets here with a size ranging from several to several tens of square meters. The plant communities of heathlands and pine forests differ mainly in their vertical structure. There are no trees or shrubs in the heathlands, while *Pinus sylvestris* dominates in the pine forests. Therefore, these two habitat types differ in, e.g., their light conditions. The soils of the analyzed bearberry populations are mainly podzols with a predominant (>90%) sand fraction. These are mostly very acidic soils with a pH (KCl) value below 4.5. In most cases, the content of organic matter does not exceed 2%. Most of the soils are characterized by a very low abundance in available forms of P_2_O_5_, K_2_O, and MgO.

In each of the 20 *Arctostaphylos uva-ursi* populations, three samples of plant material (each 40 g leaf fresh weight) were collected within 25-m^2^ compact patches (with bearberry coverage 90–100%) for the phytochemical analyses. After collection, the plant material was placed in a refrigerator and transported to the laboratory, where it was dried.

### 3.2. Preparation of Leaf Extracts

Dried leaves of bearberry were ground in a laboratory mill to pass through a 1-mm screen. Powdered plant material (0.5 g) was extracted with 50 mL of 70% ethanol in distilled water (*v*/*v*) in an ultrasonic water bath for 1 h at 40 °C. The extracts were centrifuged for 15 min at 4500× *g* and the supernatants were filtered through laboratory filter paper. The extracts were stored in a freezer at −50 °C until analysis.

### 3.3. Chemicals and Reagents

Chromatographic standards and eluents: arbutin (ARB; 4-hydroxyphenyl-β-D-glucopyranoside) (≥98%), picein (PIC; 4-acetylphenyl β-D-glucopyranoside) (≥98%), methylarbutin (mARB; 4-methoxyphenyl β-D-glucopyranoside (≥97%), corilagin (COR; 1-O-galloyl-3,6-hexahydroxydiphenol-β-D-glucopyranose) (≥96%), hyperoside (HYP; 3,3′,4′,5,7-pentahydroxyflavone 3-D-galactoside) (≥95.0%), penta-O-galloyl-β-d-glucose (PGG) (≥96%), myricetin (MYR; 3,3′,4′,5,5′,7-hexahydroxyflavone) (≥96%), acetonitrile (HPLC grade), and formic acid (HPLC grade) as well as reagents and standards for total phenolic and antioxidant assays: *Folin-Ciocalteu*’s phenol reagent, gallic acid (GA; 3,4,5-trihydroxybenzoic acid) (≥98%), aluminum chloride (AlCl_3_), quercetin (Q; 3,3′,4′,5,6-pentahydroxyflavone) (≥97%), ABTS (2,2′-azino-bis (3-ethylbenzothiazoline-6-sulphonic acid), DPPH (2,2-diphenyl-1-picrylhydrazyl), TPTZ (2,4,6-Tri(2-pyridyl)-s-triazine), Trolox (6-hydroxy-2,5,7,8-tetramethylchroman-2-carboxylic acid) (≥97%), ferrozine (3-(2-pyridyl)-5,6-diphenyl-1,2,4-triazine-4′,4′′-disulfonic acid sodium salt), EDTA (ethylenediaminetetraacetic acid disodium salt) (≥99%) were supplied by Merck company (Merck KGaA, Darmstadt, Germany). All other chemicals were of analytical grade.

### 3.4. Chromatographic Analysis of Arctostaphylos uva-ursi (L.) Phytochemicals

High-performance liquid chromatography (HPLC) was performed on a Varian ProStar HPLC separation system coupled with a ProStar 210 solvent delivery module, a ProStar 325 UV-Vis detector (Varian Inc., Walnut Creek, CA, USA), and a Gemini column (250 mm × 4.6 mm) packed with octadecyl group bonded type silica gel (C18)—pore size 110 Å, particle diameter 5 μm (Phenomenex, Torrance, CA, USA). Prior to the analysis, the extracts were diluted 10-times and filtered through a 0.2-µm syringe nylon filter. The injection volume was 20 µL. The column thermostat was set at 25 °C and elution was carried out at a flow rate of 1 mL min^−1^. Mobile phase A contained 0.1% formic acid in H_2_O and mobile phase B contained 0.1% formic acid in acetonitrile. The elution gradient mode was as follows: 4% B (pre-run), 4–22% B (0–25 min), 22–25% B (25–40 min), 25–100% B (40–50 min), 100% B (50–55 min), 100–4% B (55–60 min), 4% B (60–65 min). The chromatograms were acquired at λ = 280 nm for ARB, mARB, PIC, COR, and PGG and at λ = 350 nm for HYP and MYR. Quantification of individual phytochemicals was performed using calibration curves for standards. The results were expressed in mg g^−1^ dry matter of plant material.

### 3.5. Determination of Total Phenolic Content (TPC)

The content of total polyphenols was determined spectrophotometrically using Folin–Ciocalteu reagent [76]. Before the analysis, the bearberry extracts were diluted 25-times. The diluted extract (10 µL) was mixed with 40 µL of a Folin–Ciocalteu reagent solution in distilled water (1:5; *v*/*v*) and left to stand for 3 min at room temperature. Then, 100 µL of 10% (*v*/*v*) sodium carbonate and 150 µL of distilled water were added. After 30 min, absorbance was measured at λ = 765 nm against the blank sample using an Epoch 2 microplate spectrophotometer (BioTek Instruments, Inc., Winooski, VT, USA). Results were expressed as gallic acid equivalents (GAE) in mg g^−1^ dry matter of plant material.

### 3.6. Determination of Total Flavonoid Content (TFC)

The total flavonoid content was determined using a spectrophotometric assay following a procedure proposed by Lamaison and Carnat [77] based on the formation of complexes with aluminum chloride. Before the analysis, the extracts were diluted 5-times. The diluted extract (150 µL) was mixed with 150 µL of 3% aluminum chloride in 70% ethanol (*w*/*v*). The reaction mixture was incubated for 30 min. Then, absorbance was measured at λ = 430 nm against a blank sample using an Epoch 2 microplate spectrophotometer (BioTek Instruments, Inc.). Results were expressed as quercetin equivalents (QE) in mg g^−1^ dry matter of plant material.

### 3.7. Antioxidant Activity

#### 3.7.1. ABTS^•+^ Scavenging Activity (ABTS)

ABTS^•+^ scavenging activity was determined based on the decolourization assay according to the method developed by Re et al. [78]. ABTS^•+^ was generated by reaction with potassium persulfate strictly according to the procedure proposed by Re et al. [78]. The ABTS^•+^ stock solution was diluted with 70% ethanol (*v*/*v*) to absorbance 0.700 ± 0.01 measured for a 1-cm path length at λ = 734 nm. Before the analysis, the extracts were diluted 50-times. The diluted extract (5 µL) was mixed with 300 µL of an ethanolic solution of ABTS^•+^ and left to stand for 120 min at room temperature. Absorbance was recorded at λ = 734 nm using an Epoch 2 microplate spectrophotometer (BioTek Instruments, Inc.). Results were expressed as Trolox equivalents (TE) in mg g^−1^ dry matter of plant material.

#### 3.7.2. DPPH^•^ Scavenging Activity (DPPH)

DPPH^•^ scavenging activity was determined according to the procedure described by Brand-Williams, Cuvelier and Berset [79]. Before the analysis, the extracts were diluted 50-times. The diluted extract (5 µL) was mixed with 300 µL of 60 µM DPPH^•^ in methanol and left to stand for 120 min at room temperature. Absorbance was recorded at λ = 515 nm using an Epoch 2 microplate spectrophotometer (BioTek Instruments, Inc.). Results were expressed as Trolox equivalents (TE) in mg g^−1^ dry matter of plant material.

#### 3.7.3. Reducing Power (RP)

Ferric reducing power was determined according to the method developed by Benzie and Strain [80]. The assay reagent was prepared by mixing 0.3 M sodium acetate buffer (pH 3.6), 10 mM TPTZ, and 20 mM iron (III) chloride at a ratio of 10:1:1 (*v*/*v*/*v*), respectively. Before the analysis, the extracts were diluted 25-times. The diluted extract (10 µL) was mixed with 40 µL of the assay reagent and 200 µL of distilled water. After 30 min of incubation, absorbance was measured at λ = 593 nm against a blank sample using an Epoch 2 microplate spectrophotometer (BioTek Instruments, Inc.). Results were expressed as Trolox equivalents (TE) in mg g^−1^ dry matter of plant material.

#### 3.7.4. Chelating Ability (CHEL)

For the determination of ferrous ion chelating ability, the ferrozine assay was adapted from Guo et al. [81]. Before the analysis, the extracts were diluted 10-times. The diluted extract (200 µL) was mixed with 25 µL of 2 mM iron (II) chloride and 10 µL of 5 mM ferrozine. The absorbance of the reaction mixture was recorded after 10 min at λ = 562 nm using an Epoch 2 microplate spectrophotometer (BioTek Instruments, Inc.). Results were expressed as ethylenediaminetetraacetic acid equivalents (EDTA) in mg g^−1^ dry matter of plant material.

### 3.8. Statistical Analysis

After testing the data for normality and homoscedasticity, analysis of variance of different sets of data was performed followed by subsequent Tukey’s test or the non-parametric test as Kruskal-Wallis test, and the subsequent U Mann-Whitney test. The results were expressed as means and SD, and the differences were considered significant at *p* < 0.05. Correlations (Pearson coefficient) were estimated as well. The statistical analyses were carried out using the Statistica 6.0 software (Stat. Soft, Inc., Krakow, Poland). Principal component analysis (PCA) was applied to explain the relationships between the presented parameters and to show variability factors. Prior to the PCA, the data were centered. The analyses were carried out using the statistical package (MVSP) program version 3.1 [82].

## 4. Conclusions

The bearberry is a valuable source of phenolic compounds, which are mainly responsible for the antioxidant properties of extracts. The main phenolic compound of bearberry found in the extracts was arbutin; however, other valuable phenolic compounds such as methylarbutin, penta-O-galloyl-β-d-glucose, hyperoside, picein and corilagin were determined as well. The relationship between the content of ARB and TPC proves the high share of phenolic compounds other than arbutin. Furthermore, as reported, the locality and environmental conditions (heathlands or pine forest) affected the phytochemical profiles and antioxidant activity of the bearberry samples. For example, the extracts from bearberry leaves collected from the heathland population were characterized by significantly higher content of flavonoids (total) and hyperoside and significantly lower content of methylarbutin and corilagin in comparison with the pine forest population. In addition, the review of literature data indicates that the investigated herbal material was characterized by a different chemical profile compared to the chemical profiles of bearberry found in other parts of Europe. This study showed that the analyzed bearberry samples differed in the arbutin, miricetin, and total phenolic contents from the profile in the Iberian Peninsula population and the picein content from the Russian population. In the vast majority of cases, the bearberry leaves from the studied Polish populations meet the European Pharmacopoeia requirements regarding the content of arbutin (>70 mg g^−1^); hence, they can be classified as herbal material and used in pharmacy. Nevertheless, the high content of phenols and the high values of antioxidant parameters indicate a high potential of bearberry leaves to be used as a powerful natural source of antioxidants in herbal preparations, as potential food and cosmetics additives, and in other applications. The studied bearberry populations are characterized by a very interesting chemical profile; however, future studies are needed to determine their potential bioactivity considering the occurrence of this plant material.

## Figures and Tables

**Table 1:** Results of PCA based on the chemical composition of bearberry leaf extracts.

Variables	Axis 1	Axis 2	Axis 3
Eigenvalues	2.34	1.62	1.24
Percentage	29.28	20.19	15.51
Cumulative percentage	29.28	49.48	64.99
ARB	0.100	−0.471	−0.168
mARB	0.362	0.056	0.542
PGG	0.101	−0.439	0.403
HYP	−0.585	−0.113	0.312
PIC	−0.089	0.518	0.321
COR	0.378	0.048	0.454
TPC	0.060	−0.536	0.094
TFC	−0.593	−0.110	0.315

ARB—arbutin, mARB—methylarbutin, PGG—penta-O-galloyl-β-d-glucose, HYP—hyperoside, PIC—picein, COR—corilagin, TPC—total phenolic content, TFC—total flavonoid content.

**Table 2:** Antioxidant activity parameters of leaf extracts from bearberry plants collected in heathland populations (No 1–10) and in pine forest populations (No 11–20). For each site, data are mean and SD.

No	ABTS (mg TE g^−1^)		DPPH (mg TE g^−1^)		RP (mg TE g^−1^)		CHEL (mg EDTA g^−1^)	
	Mean	SD	Mean	SD	Mean	SD	Mean	SD
1	551.06 ^a^	5.24	420.66 ^ab^	8.88	359.85 ^ab^	2.51	3.28 ^a^	0.32
2	530.63 ^a^	12.94	374.76 ^a^	19.28	343.38 ^a^	3.51	4.02 ^bc^	0.06
3	654.66 ^c^	20.75	515.25 ^cd^	19.24	401.30 ^de^	4.33	4.08 ^bc^	0.23
4	654.34 ^c^	15.79	542.71 ^d^	23.59	393.70 ^cde^	1.57	3.41 ^a^	0.07
5	597.46 ^b^	12.09	469.43 ^b^	9.27	389.15 ^cd^	3.23	4.03 ^bc^	0.16
6	676.16 ^cd^	16.28	551.63 ^d^	7.72	365.24 ^b^	6.94	3.90 ^bc^	0.14
7	707.67 ^b^	7.11	682.17 ^e^	24.33	406.64 ^de^	8.04	3.59	0.17
8	561.95 ^ab^	12.95	541.87 ^d^	11.14	344.75 ^a^	5.67	4.34 ^ab^	0.11
9	713.49 ^d^	21.43	756.62 ^f^	24.45	408.05 ^e^	4.13	4.84 ^cd^	0.17
10	593.34 ^b^	23.31	456.75 ^b^	9.22	384.94 ^bc^	4.35	4.21 ^de^	0.09
11	652.70 ^c^	3.95	508.44 ^cd^	13.29	398.57 ^de^	7.84	4.69 ^c^	0.18
12	561.95 ^ab^	7.12	392.84 ^a^	8.85	361.46 ^ab^	8.92	4.42 ^d^	0.28
13	547.18 ^a^	3.94	377.33 ^a^	11.12	351.89 ^a^	3.05	4.60 ^d^	0.09
14	561.95 ^ab^	7.90	412.02 ^ab^	13.52	377.75 ^bc^	9.83	5.34 ^d^	0.10
15	596.31 ^b^	8.66	447.16 ^b^	21.97	383.76 ^bc^	5.58	4.61 ^cd^	0.14
16	556.75 ^a^	10.32	415.57 ^ab^	27.18	365.13 ^b^	6.00	4.82 ^de^	0.08
17	643.71 ^c^	9.91	518.34 ^d^	13.32	402.67 ^de^	5.77	4.89 ^de^	0.04
18	543.71 ^a^	9.05	372.18 ^a^	13.52	376.51 ^bc^	7.64	4.78 ^d^	0.16
19	560.28 ^ab^	8.58	401.97 ^a^	12.74	375.60 ^bc^	8.82	4.19 ^c^	0.23
20	548.77 ^a^	6.86	423.62 ^ab^	10.25	401.23 ^de^	2.51	3.56 ^ab^	0.16

ABTS—ABTS^•+^ scavenging activity, DPPH—DPPH^•^ scavenging activity, RP—reducing power, CHEL—chelating ability. The values designated by the different letters (a–f) are significantly different (*p* < 0.05).

**Table 3:** Results of PCA based on the chemical composition of bearberry leaf extracts and antioxidant activity parameters.

Variables	Axis 1	Axis 2	Axis 3
Eigenvalues	3.38	2.48	1.60
Percentage	28.17	20.65	13.32
Cumulative percentage	28.17	48.83	62.15
ARB	0.186	0.143	−0.596
mARB	0.045	0.351	0.347
PGG	−0.052	0.237	−0.22
HYP	−0.346	−0.35	−0.195
PIC	0.036	−0.204	0.424
COR	0.156	0.304	0.264
TPC	−0.277	0.402	−0.267
TFC	−0.353	−0.359	−0.181
ABTS	−0.493	0.134	0.067
DPPH	−0.488	0.034	0.244
RP	−0.34	0.441	0.018
CHEL	0.132	0.198	−0.146

ARB—arbutin, mARB—methylarbutin, PGG—penta-O-galloyl-β-d-glucose, HYP—hyperoside, PIC—picein, COR—corilagin, TPC—total phenolic content, TFC—total flavonoid content, ABTS—ABTS^•+^ scavenging activity, DPPH—DPPH^•^ scavenging activity, RP—reducing power, CHEL—chelating ability.

**Table 4:** Correlation coefficients for relationships between the studied secondary metabolites in extracts of bearberry leaves from the heathlands and antioxidant parameters (*n* = 30); bolded correlation coefficients are statistically significant. Explanations see Table 3.

	ABTS	DPPH	RP	CHEL
**ARB**	−0.236	−0.586 **	0.041	−0.090
**mARB**	0.537 **	0.587 ***	0.240	0.205
**PGG**	0.005	0.093	0.132	0.597 **
**HYP**	0.151	0.076	0.003	0.410 *
**PIC**	−0.144	−0.001	−0.202	−0.004
**COR**	0.108	0.457	0.247	0.515 **
**TPC**	0.517 **	0.239	0.859 ***	−0.076
**TFC**	0.054	0.073	0.035	0.395 *

* *p* < 0.05; ** *p* < 0.01; *** *p* < 0.001.

**Table 5:** Correlation coefficients for relationships between the studied secondary metabolites in extracts of bearberry leaves from the pine forests and antioxidant parameters (*n* = 30); bolded correlation coefficients are statistically significant. Explanations see Table 3.

	ABTS	DPPH	RP	CHEL
**ARB**	−0.291	−0.456 *	−0.202	0.220
**mARB**	0.094	0.103	0.229	−0.390 *
**PGG**	0.370 *	0.349	0.221	0.014
**HYP**	0.137	−0.015	0.104	0.561 **
**PIC**	−0.096	−0.128	0.236	−0.424 *
**COR**	−0.398 *	−0.289	0.331	−0.587 **
**TPC**	0.641 ***	0.690 ***	0.857 ***	0.014
**TFC**	0.019	−0.019	0.065	0.634 ***

* *p* < 0.05; ** *p* < 0.01; *** *p* < 0.001.

## Sample Availability

Samples are available from the authors.
